# Oral Spirulina Platensis Attenuates Hyperglycemia and Exhibits Antinociceptive Effect in Streptozotocin-Induced Diabetic Neuropathy Rat Model

**DOI:** 10.2147/JPR.S267347

**Published:** 2020-09-15

**Authors:** Mohamed M Abdel-Daim, Mohamed Shaaban Ali, Fedekar F Madkour, Hamed Elgendy

**Affiliations:** 1 Pharmacology Department, Faculty of Veterinary Medicine, Suez Canal University, Ismailia 41522, Egypt; 2 Anesthesia Department, Assiut University Hospitals, Assiut, Egypt; 3 Marine Science Department, Faculty of Science, Port Said University, Port Said 42526, Egypt; 4 Anesthesia Department, HAMAD Medical Corporation & Weill Cornell Medicine Qatar & Qatar University, Doha, Qatar

**Keywords:** antinociceptive, *Spirulina platensis*, diabetes, neuropathy, rat

## Abstract

**Introduction:**

Diabetic neuropathy is a common consequence of diabetes. Hyperalgesia is one of the main symptoms of diabetic neuropathy. In response to noxious stimuli, streptozotocin (STZ)-induced diabetic rats show exaggerated hyperalgesic behavior, while Spirulina platensis has anti-inflammatory, antioxidant, and insulin-like effects. To assess the antinociceptive effect of oral *Spirulina platensis* (SP) powder on formalin-induced nociceptive responses in STZ-induced diabetic rats.

**Methods:**

Sixty mature male *albino* rats were randomly allocated into six equal groups (10 in each group). Group 1 (control non-diabetic group) received 0.9% saline; group 2 was given oral pure SP powder-treated as a non-diabetic control group, group 3 was sodium salicylate-treated rats and used as a positive non-diabetic control group, group 4 managed as vehicle-treated diabetic rats, group 5 considered as SP-treated-diabetic group, and sodium salicylate-treated-diabetic rats used as a diabetic positive control group (group 6). STZ-diabetic rats were orally given SP in a dose of 500 mg kg/day for 1 month. The formalin test was implemented in two phases: the early phase in the first 10-min post-formalin injection, and the late phase was considered in the 15–60 min post-formalin injection time interval.

**Results:**

Pain scores were increased in the diabetic groups during both phases of the experiment. Blood glucose was significantly reduced in diabetic rats that received oral SP, P < 0.01. Besides, SP-treated rats had lower pain scores during both phases of the experiment than untreated diabetic ones. However, in the sodium salicylate group, the pain score was reduced only during the second phase. An exaggerated nociceptive response occurred in diabetic rats after the formalin test. A significant antinociceptive effect appeared in SP-treated control and diabetic rats.

**Discussion:**

The findings suggest that oral *Spirulina platensis* could have a potential therapeutic role for managing induced painful diabetic neuropathy in rats.

## Introduction

Diabetes is a common chronic disabling disease resulting in chronic hyperglycemia that produces perturbation of cellular metabolism. It disturbs the metabolic pathways, leads to excessive free radical production and oxidative stress. Hyperglycemia, either acute or chronic, leads to oxidative stress and microvascular consequences in the peripheral nerves and initiates diabetic neuropathy. Diabetic neuropathy is a common microvascular disease that estimated recently, approximately 10–20% in diabetic subjects who exhibit painful neuropathy.^1^

Oxidative stress process occurs when a cellular system fails to get rid of the accumulated oxygen free radicals produced during metabolism. These radicals attack and destruct proteins, lipids, and nucleic acids, contributing to disturbance of energy metabolism, cell signaling, and transport activities. These accumulated oxygen-free radicals lead to cell death through programmed cell death pathway.^2^

Acute hyperglycemia in type-2 diabetes is also closely linked to reactive oxygen species-mediated oxidative stress.^3^ Numerous dietary supplements were tried in different countries as a traditional food that thought to be beneficial and activates the total radical antioxidant potentials in diabetic patients.^4^

*Spirulina platensis* (SP) is naturally growing freshwater blue-green algae, enriched with proteins and essential nutrients. The use of SP has been found to reduce high serum lipids^5^ and decrease high blood pressure.^5^,^6^ Moreover, it demonstrated neuroprotective and reactive oxygen species (ROS) scavenging actions observed in various experimental studies.^7^,^8^ A biliprotein, C-phycocyanin, extracted from SP, had anti-inflammatory activity.^9^ Antioxidant properties of SP were described before.^10^,^11^

Jung et al extracted insulin-like protein from SP with the same molecular mass, immunoreactivity, as that of bovine insulin. It has been successfully decreased diabetic complications by increasing body weight and markedly reducing high blood glucose and glycosylated hemoglobin in diabetic rats.^12^ It showed improved glycemic control as consistent with another experimental study.^13^

Up to our knowledge, *Spirulina platensis* is not investigated before in the treatment of painful diabetic neuropathy. Therefore, we initiated our rat model of formalin-induced painful diabetic neuropathy. To evaluate the oral administration of 500 mg/kg SP for 1 month for attenuation of diabetes and antinociceptive effect on painful diabetic neuropathic rat model.

## Materials and Methods

We have the approval of our Institutional Board Ethical Committee for animal research at the Faculty of Veterinary Medicine, Suez Canal University, on 06-11-2018 (Registration no. 14–186). We performed all the procedures, and rats were treated in conformity with and followed the principles on ethical animal research outlined in the ethical guidelines by the International Council for Laboratory Animal Science.

### Chemicals

*Spirulina platensis* powder in a pure premium form was obtained from (Herba Force, UK). The strain of *Spirulina platensis* was purchased from CFTRI Mysore, India, as used in a previous study.^14^

### Animals

**Figure 1:**
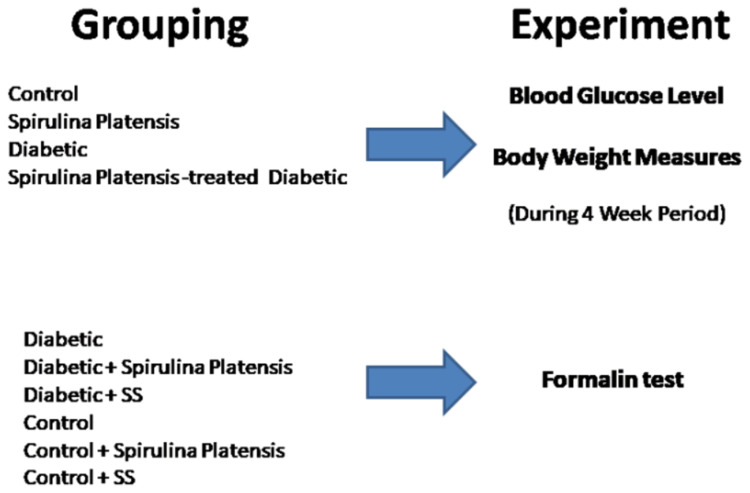
Algorithm of the study design.

**Abbreviation:** SS, sodium salicylate.

Mature male *albino* rats weighing 180–230 g were purchased from the animal house of National Research Centre, Dokki-Cairo. The animals were housed individually in cages, kept on a 12 h light/dark cycle, at a comfortable temperature (21 ± 1°C), humidity (55 ± 5%), and free access to food and water at all times. We demonstrated our experimental model in Figure 1. Sixty mature male *albino* rats were randomly allocated into six equal groups (10 in each group). Group 1 (control non-diabetic group) received 0.9% saline; group 2 was given oral pure SP powder-treated as the non-diabetic control group, group 3 was sodium salicylate-managed rats and used as a positive non-diabetic control group, group 4 managed as vehicle-treated diabetic rats, group 5 considered as SP-treated-diabetic group, and sodium salicylate-treated- diabetic animals used as a diabetic positive control group (group 6). STZ-diabetic rats were orally given SP using a stomach tube in a dose of 500 mg kg/day for 1 month. We tested this dose before in a previous study of our group.^14^

### Induction of Diabetes

To ensure that streptozotocin (STZ) have injured islets of langerhans, 30 rats were kept on overnight fasting before the STZ injection, as fasting could induce quick stress injury to islets as reported by Zhang et al.^15^ STZ was freshly prepared by dissolving 50 mM sodium citrate (pH 4.5) solution containing 150 mM NaCl, which was administered as a subcutaneous injection at the dose of 60 mg/kg body weight. The administration of STZ should be done immediately after fresh preparation. Blood glucose level was measured by electronic glucometer. After the STZ injection by 2 weeks, we confirmed that those animals developed maintained hyperglycemia above 400 mg/dL and then started to perform data analysis.^16^

### Formalin Test

For pain assessment, the formalin test was performed, which was described elsewhere.^17^ A summary, before starting the test, each rat was acclimatized to the observation box.

Fifty microlitre of 2.5% formalin was injected subcutaneously, in the planter surface of right-hand paw, using a 25-gauge syringe needle. Immediately, each experimental animal was placed in a Plexiglas box. First, we began determining nociceptive responses by placing each animal in the box and continued observations for 60 min. We could determine the nociceptive score for each 5 min block during that period by time measurement, which was spent in each of the four behavioral categories: 0 nociceptive score when the position and posture of the injected hind paw are indistinguishable from the contralateral paw. Level 1 determined when the injected paw has little or no weight placed on it. Level number 2 could be distinguished when the injected paw is elevated and is not in contact with any surface. The third nociceptive score level could be determined when the injected paw is licked, bitten, or shaken. We have measured a weighted nociceptive score, which ranged from 0 to 3, was calculated by multiplying the time spent in each category by the category weight, summing these products, and dividing by the total time for each 5 min block of time. Two phases were determined; the early (first) phase in the first 10-min post-formalin injection, and the late (second) phase was considered in the time interval 15–60-min post-formalin injection.^18^

### Study Design

*Spirulina platensis* powder was administered orally using a stomach tube; we used 500 mg/kg for 1 month starting from the day 15th after STZ administration for the second and fifth groups. Sodium salicylate was administered orally for the third and sixth groups at a dose of 200 mg/kg 60 min before the formalin test. Body weight and serum blood glucose levels for all rats were monitored at the beginning and the end of the experiment. After 8 h of fasting, serial blood samples were collected by tail nipping and assessed for blood glucose by an electronic portable glucometer.

### Data and Statistical Analysis

The normality of data was checked by Shapiro–Wilk’s test. Data on blood glucose level and pain score in formalin test showed normal distribution P-value >0.05; hence, data were expressed as mean ± SD and groups were compared using student’s paired *t*-test. While data of weight showed P-value <0.05 using Shapiro–Wilk’s test (did not show the normality of data) and expressed as mean ± S.E.M. and analyzed using nonparametric test; Wilcoxon Signed Ranks Test. The probability level at P ≤ 0.05 is considered Significant. Analysis was done on SPSS (IBM Inc., the USA) version 21.0.

## Results

Figure 1 demonstrates the algorithm of the study design. Besides, serial blood glucose measurements before and after treatment are described in Table 1.

**Table 1:** Effect of Spirulina on Blood Glucose Level

Group Blood Glucose mg/dl	Control	Spirulina	Diabetic	Spirulina-Treated Diabetic
0 week (mean ± SD)	90.5 ± 9.11	89.1 ± 7.43	90.7 ± 6.8	89.9 ± 6.37
4th week	91.3 ± 6.72	87.6 ± 7.01	258.6 ± 27.59	154 ± 26.12
*P -* value	0.665	0.241	< 0.001	< 0.01

**Notes:** Using student’s Paired *T*-test, *P* ˂ 0.05 considered statistically significant.

**Abbreviations**: mg/dl, milligram per decilitre; SD, standard deviation.

The first and second groups showed average blood glucose values below 100 mg/dl before and after a month of treatment. The third vehicle-treated diabetic group showed a significant rise in blood glucose after 1 month above 250 mg/dl (P ˂ 0.001). However, the fourth Spirulina platensis oral powder-treated diabetic group revealed better control of blood sugar to below 150 mg/dl (P ˂ 0.01), as shown in Table 1.

During the blood glucose measuring experiment, we observed a significant decrease in blood glucose in diabetic rats with *Spirulina* compared with the diabetic non-treated group, as shown in Table 1.

Regarding Body weight gain in our examined rats, the *Spirulina platensis*-treated diabetic group had gained weight after 4 weeks, (*P =* 0.066), as well values of the control *Spirulina platensis*-treated group showed (*P =* 0.028). However, the third vehicle-treated diabetic group showed insignificant weight gain (*P =* 0.332) as shown in Table 2.

**Table 2:** Effect of Spirulina on Weight Gain

Group Weight per Gram	Control	Spirulina	Diabetic	Spirulina-Treated Diabetic
0 week (mean ± SE)	199.3 ± 4	199.1 ± 3.4	199.2 ± 4	201.3 ± 4.2
4th week	211.3 ± 4.2	215.1 ± 3.7	204.8 ± 4	214.6 ± 4
*P-* value	0.103	0.028	0.332	0.066

**Notes:** Using Wilcoxon Signed Ranks Test (nonparametric). *P* ˂ 0.05 considered statistically significant.

**Abbreviation:** SE, standard error.

During the formalin test experiment, we observed that the diabetic rats’ group showed an elevated pain score in both phases. We observed a reduced level of pain score for both formalin test phases in *Spirulina platensis* - received diabetic rats in comparison to untreated-diabetic animals, P < 0.01. Also, SP-treated rats had lower pain scores during both phases of the experiment than untreated diabetic ones.

**Figure 2:**
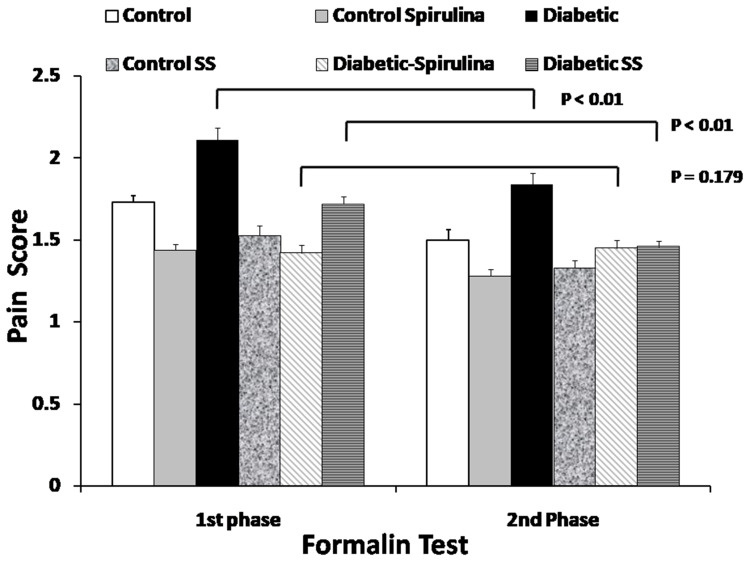
The effect of oral Spirulina Platensis and sodium salicylate on nociceptive scores in 1st (early) and 2nd (late) phases of formalin test. Six groups were tested: Control; Control + Spirulina Platensis; Diabetic; Control + SS; Diabetic + Spirulina Platensis and Diabetic + SS. Spirulina Platensis - treated diabetic rats showed a lower pain score for both phases of formalin test (P= 0.179) as compared to untreated-diabetic animals, P < 0.01.

**Abbreviation:** SS, sodium salicylate.

However, in the sodium salicylate (positive control) group, the pain score was reduced only during the second phase. At the same time, an exaggerated nociceptive response occurred in diabetic rats after the formalin test. A significant antinociceptive effect appeared in SP-treated control and diabetic rats, as shown in Figure 2.

## Discussion

The present study revealed that the administration of *Spirulina platensis* orally could attenuate diabetes with improved blood glucose levels and weight gain. Implementation of the formalin test demonstrated a pronounced antinociceptive response in control and diabetic rats at both phases of that test.

*Spirulina platensis* has many health benefits: antioxidant advantages,^10^ anti-inflammatory activity,^9^ anti-diabetic actions,^12^,^13^ and hypocholesterolemic.^19^

Pain is categorized depending on its cause, as the first type of nociceptive, second type as neuropathic or mixed of both forms.^20^ Different modalities of treating diabetic neuropathy were described in animal models. Anticonvulsants, especially gabapentin and carbamazepine, and opioids were ultimately used.^21^

Regarding its anti-inflammatory characteristics, Shih et al^9^ had reported that C-phycocyanin, which was an extract of *Spirulina platensis*, revealed anti-inﬂammatory and anti-hyperalgesic effects. Another experimental study demonstrated a significant decrease in inflammatory mediators’ activities, for instance, IL-6 and IL-1β caused by *Spirulina platensis*. A mouse model of zymosan-induced arthritis explored this anti-inflammatory activity.^22^

Chen et al^23^ also demonstrated: the microglial cells exhibited enormous anti-inflammatory activity of phycocyanin material and *Spirulina*. They concluded that both materials significantly inhibited the release of lipopolysaccharide-induced lactate dehydrogenase. They suppressed inflammatory mediators’ expression, for instance, iNOS, IL-6, COX-2, and TNF-α.

Regarding the anti-diabetic effect of *Spirulina platensis*, we think that the insulin-like effect of *Spirulina platensis* may be responsible for diabetic state attenuation of the diabetic treated rats as consistent with previous studies.^5^,^13^ Li et al discovered that Spirulina protects pancreatic Beta cells, which could explain blood glucose control.^24^ Moreover, Spirulina’s active materials include immunoreactive insulin; therefore, diabetic subjects may get a crucial benefit.^25^ In our model attenuation of diabetic state, we observed weight gain in *Spirulina platensis*-treated diabetic rats.

*Spirulina platensis* antioxidant effect was reported before.^11^ Usage of Spirulina as a nutritional supplement a long time ago was attributed to its structure as filamentous cyanobacteria.^26^ Phycocyanin is one of the significant constituents of *Spirulina platensis*. The tetrapyrrole chromophore gives its deep blue color of phycocyanin. Moreover, phycocyanobilin is covalently connected to the apoprotein. The phycocyanin conveys inhibition of oxygen free radical production. *Spirulina platensis* attributes its promising antioxidant properties to phycocyanobilin.^26^

Spirulina antioxidant properties can be activated through various pathways. First is through activation of the scavenging system, and secondly, it is by preventing DNA damage and lipid peroxidation. Moreover, it augments the activity of superoxide dismutase, catalase, and suppresses reactive oxygen species.^27^ It was proved that Spirulina organizes the IκB signaling as well the p38 pathways to exhibit its free radicals scavenging, anti-inflammatory and immunomodulatory properties.^28^

Treatment with Spirulina significantly inhibits oxygen free radicals as many experimental studies reported it. These protective effects may be attributed to phycocyanins, β-carotene, and other substances included within Spirulina.^14^

One study exposed neuroblastoma cells to oxidative stress injury and explored *Spirulina platensis* suppress lipid peroxidation. Bermejo-Bescóset et al discussed the impact of Spirulina antioxidant activity on glutathione reduction and peroxidation enzymes.^11^

Interestingly, when additional environmental stress factor is excited, the protective ability of *Spirulina platensis* could be boosted.^27^ Therefore, these promising algae could be crucial agents for the management of disorders aggravated by reactive oxygen species and the creation of novel treatment evolution.

Moreover, Spirulina safeguards against neurotoxicity in rats by inhibiting oxygen free radicals.^14^ Neurotoxicity rat – model was induced using 6-hydroxydopamine and revealed the antagonizing effect of Spirulina. As consistent with our study, which showed a reduction in diabetes mellitus induced neuropathy in a painful rat model.^29^

Schmeichel^30^ et al demonstrated that an imbalance between reactive oxygen species and its detoxification in chronic hyperglycemia could be crucial for neuropathy in animal models. Moreover, neuronal cell damage in diabetes is guided by programmed cell death through the unifying mechanism by supporting glial cells.^31^

Production of oxygen free radicals influenced mitochondrial dysfunction, which contributes markedly to neuropathy.^32^ On the other hand, programmed cell death of the peripheral neurons results from sudden glycemic deprivation and is expected to be through oxidative stress mechanism.^33^

Chronic hyperglycemia does not only induce neuron injury but impairs the small-caliber neurons to grow and regenerate. Diabetic neuropathy in those subjects is an enormously dynamic process and moves simultaneously towards both edges of degeneration and regeneration. Moreover, targets of any therapeutic regimens for neuropathy disorder are creating equilibrium on the side of regeneration and avoid dysregulation of this balance.^34^,^35^ Russell et al^31^ have encouraged this hypothesis, including the measurement of ROS mediators in neurons of sensory origin. He illustrated using antioxidants in the prevention of neuron injury. Moreover, Spirulina and C-phycocyanin inhibit toxic effects and inflammatory genes expression of neuronal supporting cells which explains the reduction of neurotoxicity in neuronal cells.^23^ We observed the anti-nociception effect of *Spirulina platensis* in our diabetic neuropathic rat model.

Regarding treating neurotoxicity, a study of Hwang et al demonstrated^36^ that salicylate administration results in a marked rise in IL-1β mRNAs expression, TNF-α as well as NMDA receptor in the inner ear neurons of mice. Use of Spirulina as a dietary supplementation significantly decreased the toxicity-induced tinnitus. Moreover, it suppresses the mRNAs expression of the previous inflammatory mediators in their mouse model.

Further proof comes from the dietary supplementation of mice with a hyperglycemic diet. In this stressful condition, the animals develop hyperglycemia that induces oxygen-free radicals production and impairs scavenging.^37^ This poor glycemic control worsens the disease progression and augments consequences.^38^

If we can use nutritional supplement potential like *Spirulina platensis*, which attenuate elevated blood glucose levels,^5^ potentiates anti-inflammatory,^7^ antihyperalgesic,^9^ and induce antioxidants effects against oxygen free radicals.^11^ It will be our most promising approach by using these microalgae to fight neuropathy, attenuate diabetic state, as well as other miserable consequences in diabetes as neuropathy.

In the field of Spirulina researches, there are enormous experimental studies, hoverer, few human studies have been established to examine its anti-inflammatory abilities or antioxidant properties.

A volunteer human study in the elderly populations revealed the impact of antioxidant activities on their immunological parameters.^39^ Park et al demonstrated that using dietary supplementation of Spirulina evoked a striking increase in the level of interleukin-2, while simultaneously decreasing IL-6 levels. Moreover, researchers observed a progressive increase in superoxide dismutase enzyme activity is proportional to a time-dependent change in the antioxidant level. Spirulina was tested in Diabetes Mellitus Type II patients, in terms of lowering lipid profile. However, in those patients, oral Spirulina did not affect glucose.

On the other hand, another clinical study investigated diabetic patients and followed their HbA1C and serum lipids, which revealed a marked reduction of these variables after Spirulina’s consumption for a few months.^5^ Shetty et al, when used Spirulina as an adjuvant antioxidant and implement its use in patients with oral submucous fibrosis, succeed in getting improvement.^40^

Other studies revealed that muscle destruction produced by oxidative stress could be inhibited after Spirulina administration.^41^,^42^ Their results demonstrated that MDA levels were markedly inhibited, while superoxide dismutase enzyme levels were significantly enhanced in those subjects who had taken Spirulina.

Because of Spirulina dietary supplementation is not completely established as a potential therapy, more clinical trials are warranted. Moreover, the antioxidant advantages of painful diabetic neuropathy were not touched, as we examined in our experimental model.

One of our limitations in this experimental study that we did not measure reactive species in neurons of either subject with diabetes treated and diabetic non-treated rats with *Spirulina platensis*. Therefore, we recommend a future comprehensive study, including serum and neuronal level of ROS after administration of Spirulina to our painful neuropathy rat model as a preliminary step before the human study.

## Conclusion

The results of the present study demonstrated attenuated diabetic status and antinociceptive effect of orally administrated *Spirulina platensis* for 1 month to the painful diabetic neuropathic rat model. The biochemical measurement of ROS in a future study is warranted.
